# Genome-wide analysis of serine carboxypeptidase-like protein (SCPL) family and functional validation of *Gh_SCPL42* unchromosome conferring cotton Verticillium der Verticillium wilt stress in *Gossypium hirsutum*

**DOI:** 10.1186/s12870-022-03804-5

**Published:** 2022-09-01

**Authors:** Yuxiang Wang, Jieyin Zhao, Xiaojuan Deng, Peng Wang, Shiwei Geng, Wenju Gao, Peipei Guo, Quanjia Chen, Chunping Li, Yanying Qu

**Affiliations:** 1 Engineering Research Centre of Cotton, Ministry of Education/College of Agriculture, Xinjiang Agricultural University, 311 Nongda East Road, Urumqi, 830052 China; 2 Institute of Cash Crops, Xinjiang Academy of Agricultural Sciences, Urumqi, 830052 China

**Keywords:** *Gossypium hirsutum*, SCPL gene family, Verticillium wilt, Expression analysis, VIGS

## Abstract

**Background:**

Serine carboxypeptidase-like protein (SCPL) plays an important role in response to stress in plant. However, our knowledge of the function of the SCPL gene family is limited.

**Results:**

In this study, a comprehensive and systematic analysis of SCPL gene family was conducted to explore the phylogeny and evolution of the SCPL gene in *Gossypium hirsutum*. The phenotype and molecular mechanism of silencing of the *Gh_SCPL42* under Verticillium wilt stress was also studied. Our results showed that 96 SCPL genes were observed in genome of *G. hirsutum*, which distributed on 25 chromosomes and most of them were located in the nucleus. The phylogenetic tree analysis showed that members of SCPL gene family can be divided into three subgroups in *G. hirsutum*, which are relatively conservative in evolution. SCPL gene has a wide range of tissue expression types in *G. hirsutum*. Promoter analysis showed that the most cis-acting elements related to MeJA and ABA were contained. Through RNA-seq combined with genotyping, it was found that 11 GhSCPL genes not only had significant expression changes during Verticillium wilt stress but also had differential SNPs in the upstream, downstream, exonic or intronic regions. The expression of these 11 genes in the resistant (Zhongzhimian 2) and susceptible (Junmian 1) materials was further analyzed by qRT-PCR, it was found that 6 genes showed significant expression differences in the two materials. Among them, *Gh_SCPL42* has the most obvious expression change. Furthermore, virus-induced gene silencing (VIGS) showed necrosis and yellowing of leaves and significantly higher disease severity index (DSI) and disease severity rate (DSR) values in VIGS plants than in control silenced *Gh_SCPL42* plants. Moreover, the expression levels of genes related to the SA and JA pathways were significantly downregulated. These results show that Gh_SCPL42 might improve resistance to Verticillium wilt through the SA and JA pathways in *G. hirsutum*.

**Conclusion:**

In conclusion, our findings indicated that *Gh_SCPL42* gene plays an important role in resistance to Verticillium wilt in cotton. It was provided an important theoretical basis for further research on the function of SCPL gene family and the molecular mechanism of resistance to Verticillium wilt in cotton.

**Supplementary Information:**

The online version contains supplementary material available at 10.1186/s12870-022-03804-5.

## Background

Various diseases seriously threaten cotton production [1–3]. Verticillium wilt, caused by *Verticillium dahliae*, is one the most serious diseases during cotton development [1, 3]. In recent years, with the improvement of sequencing technology and the reduction of sequencing costs, cotton genome has been continuously improved and updated, laying a foundation for the study of gene families at the genome-wide level [4–7].

SCPL protein, with PF00450 as the representative domain in Pfam. It is widely found in higher plants [8–10]. Similar structure and function were observed in SCPL proteins and serine carboxypeptidase (SCP) both from S10 family of SC carboxypeptidase. They are composed of the tertiary structure of α/β hydrolase with unique catalytic center and highly conserved topology [8]. The structure of SCPL proteins are not much different from SCP, both containing a signal peptide for cellular endocrine and transport, 4 substrate binding sites, multiple N-glycosylation sites, and evolutionarily conserved regions related to catalysis [9, 10]. These regions are generally located in the middle of the SCPL/SCP proteins, the amino acid residues are relatively conserved. However, their N-terminal and C-terminal amino acid residues are poorly conserved. Two special structures are contained in SCPL/SCP protein, one is a conserved catalytic triad (Ser-Asp-His) formed by serine (surrounded by the conserved Gly-X-Ser-X-Gly sequence, where Gly represents glycine, Ser represents serine, and X represents any amino acid), aspartic acid (Asp) and histidine (His), which can bind substrates and stabilize the reaction intermediates of the substrate-enzyme complex [9, 10]. Functionally, SCPL/SCP proteins generally have peptidase activity; while some SCPL proteins have acyltransferase activity, which can use O-glucose acyl group (1-O-β-glucose ester) as a substrate for acylation [11, 12].

SCPL proteins play vital roles in stress response and disease resistance in plant [13, 14]. A gene type I SCP was found in tomato as one of the “ late injury-inducible genes” which induced by injury, systemin and methyl jasmonate (MeJA) [15]. *OsBISCPL1* gene was significantly overexpressed in rice leaves treated with defense-related signaling molecules such as salicylic acid (SA) and jasmonic acid (JA) or infected with pathogenic bacteria [16]. Overexpressing *OsBISCPL1* in *Arabidopsis thaliana* also showed strong tolerance to pathogen infection, suggested that this gene might be involved in defense oxidative stress and pathogen infection [16]. Furthermore, SCPL proteins also have many important functions in the process of plant growth and development. Research showed gene *GS5* was crucial for grain size in rice, while the SCP46 gene was an important factor for regulating seed growth and development, which might be involved in abscisic acid signal transduction [17, 18]. The gene PsCP of SCP in pea was induced by gibberellin, involved in the early stages of reproductive and vegetative development [19]. In addition, *SCP1* and *SCP2* controlled cell development in tobacco [20]. SCPL proteins were involved in various abiotic stresses, including drought, salinity, light, nitrogen and phosphorus deficiency, etc. [21, 22].

Genome-wide analysis of the SCPL gene family has been studied in a variety of plants. 71 SCPL genes were identified in rice, 54 in *Arabidopsis thaliana*s, 57 in poplar, 47 in tea, and 210 in wheat [8, 11, 23, 24]. Therefore, it is of great significance to study SCPL gene in cotton. In our study, a genome-wide analysis of the SCPL gene family in *G. hirsutum* was performed, and 96 SCPL genes were identified. To elucidate the evolution and function of SCPL genes, phylogenetic analysis were performed and their physical location in different chromosomes were determined, gene structure, expression analysis, genotyping and VIGS functional verification were used. Our findings provided insights to understand the evolution of the SCPL gene and its role in the regulation of response to Verticillium wilt stress in *G. hirsutum*.

## Result

### Identification of the cotton SCPL gene family

In order to systematically study the copy number changes of SCPL gene family during cotton evolution. Firstly, SCPL genes were comprehensively searched from the *G. arboreum*, *G. raimondii*, *G. hirsutum* and *G. barbadense* genomes using HMMsearch. The search results were validated in the NCBI-CDD database (Fig. S1). Finally, 59, 53, 96 and 98 sequences were found in *G. arboreum*, *G. raimondii*, *G. hirsutum* and *G. barbadense*, respectively. We named them *Gh_SCPL1* ~ *Gh_SCPL96* according to their chromosomal positions of the 96 sequences in *G. hirsutum*. The length of the open reading frame (OFR) of the SCPL family gene is 175–2709 bp in *G. hirsutum*, the encoded protein contains 57–902 amino acid residues. The relative molecular mass is 6330.3–101,386.6 Da, and the theoretical isoelectric point is 4.58–10.16. Subcellular localization of the proteins showed that 62 localized in the outerMembrane, 14 in the cytoplasmic, 11 in the periplasmic and 9 in the extracellular (Table S1).

**Fig. 1:**
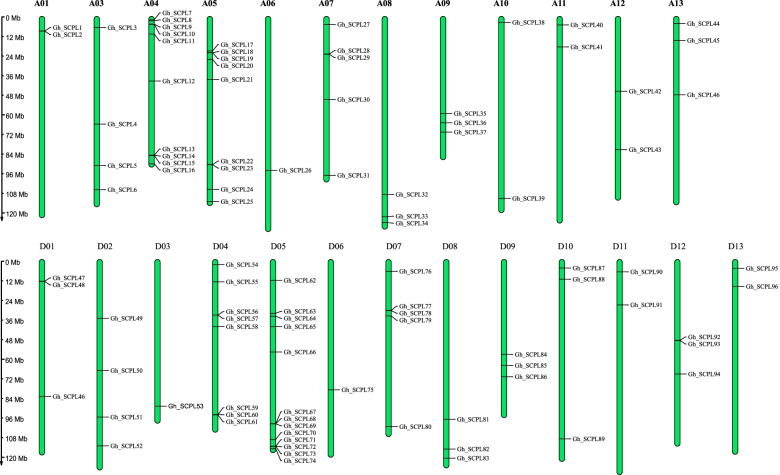
Chromosome location of SCPL genes in *G.hirsutum*

96 *GhSCPL* genes were distributed in 25 chromosomes in *G. hirsutum* (A01, A03, A04, A05, A06, A07, A08, A09, A10, A11, A12, A13, D01, D02, D03, D04, D05, D06, D07, D08, D09, D10, D11, D12, D13) (Fig. 1). Among them, 46 SCPL genes were contained in subgroup A, subgroup D contained 50 SCPL genes. Previous studies considered *G. arboreum* and *G. raimondii* to be the donor species of subgenome A and subgenome D, respectively. The number of SCPL genes in subgenome A is 13 less than that of *G. arboreum*, and the number of SCPL genes in subgenome D is 3 less than that in *G. raimondii*. It indicated that the loss of SCPL gene might have occurred due to the redundancy of gene function during the cotton evolution. There had no sequences of this family observed on A02 chromosome in *G. hirsutum*, while 4 sequences were contained in the D02 chromosome. 4, 10, 9, 2 and 3 sequences were contained in chromosomes A03, A04, A05, A12 and A13, respectively. However, only 1, 8, 13, 3 and 2 sequences of this family genes were contained in D03, D04, D05, D12 and D13 chromosomes. It showed that the SCPL gene might have lost and duplicated in the process of evolution in *G. hirsutum*. It revealed a strong correspondence between the subgenome A and the subgenome D as a whole, which was also in line with the evolutionary relationship in cotton.

### Evolutionary analysis of SCPL gene in cotton

**Fig. 2:**
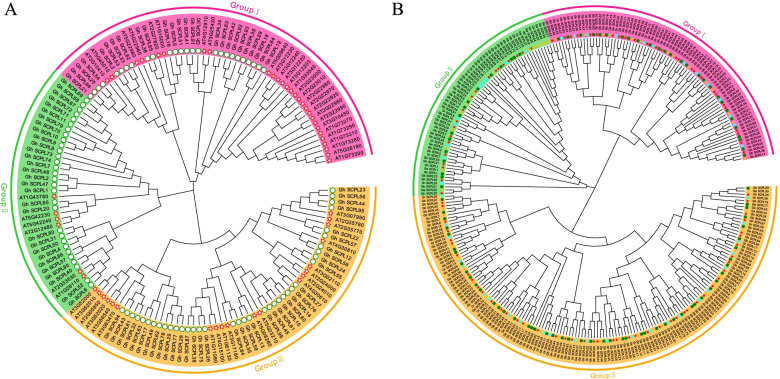
**A** shows the phylogenetic tree of *Arabidopsis thaliana* and *G.hirsutum*, The five-pointed star represents the SCPL gene of *Arabidopsis thaliana*, and the circle represents the SCPL gene of *G. hirsutum*. **B** shows the phylogenetic tree of *G. arboreum*, *G. raimondii*, *G.hirsutum*, and *G.barbadense*, The triangle represents the SCPL gene of *G. arboreum*, the circle represents the SCPL gene of *G. raimondii*, the five-pointed star represents the SCPL gene of *G. hirsutum*, and the square represents the SCPL gene of *G. barbadense*

To explore the evolutionary relationship of SCPL in cotton, a phylogenetic tree was constructed using the full-length sequences of 94 SCPL proteins from *G. hirsutum* and 54 SCPL protein sequences from *Arabidopsis thaliana* (Fig. 2A, Table S2). However, the *Gh_SCPL46* and *Gh_SCPL88* sequences were too short, they were finally deleted. According to the grouping of *Arabidopsis thaliana*, the evolutionary tree was divided into three groups. There are 26, 30 and 38 SCPL genes in I, II and III subgroups in *G. hirsutum*, respectively. The number of SCPL genes in I subgroup in *G. hirsutum* was basically the same as that of *Arabidopsis thaliana*, but it was 5 times that of Arabidopsis in II subgroup. The III subgroup was twice that of *Arabidopsis thaliana*.

In order to further understand the phylogenetic relationship of the SCPL gene family in cotton, an evolutionary tree was constructed using the protein sequences of SCPL genes from four different cotton species (Fig. 2B). The number of SCPL genes in subgroups in *G. hirsutum* and *G. barbadense* were basically twice that of *G. arboreum*, *G. raimondii*. It was consistent with the results of the previous analysis. It showed that SCPL family genes were relatively conserved in the evolution of cotton. Although groups I and II had relatively few members, they have been retained during the evolution of cotton, suggesting that they might play vital roles in biological processes.

### Evolutionary tree, gene structure and motif analysis of SCPL gene in *G. hirsutum*

**Fig. 3:**
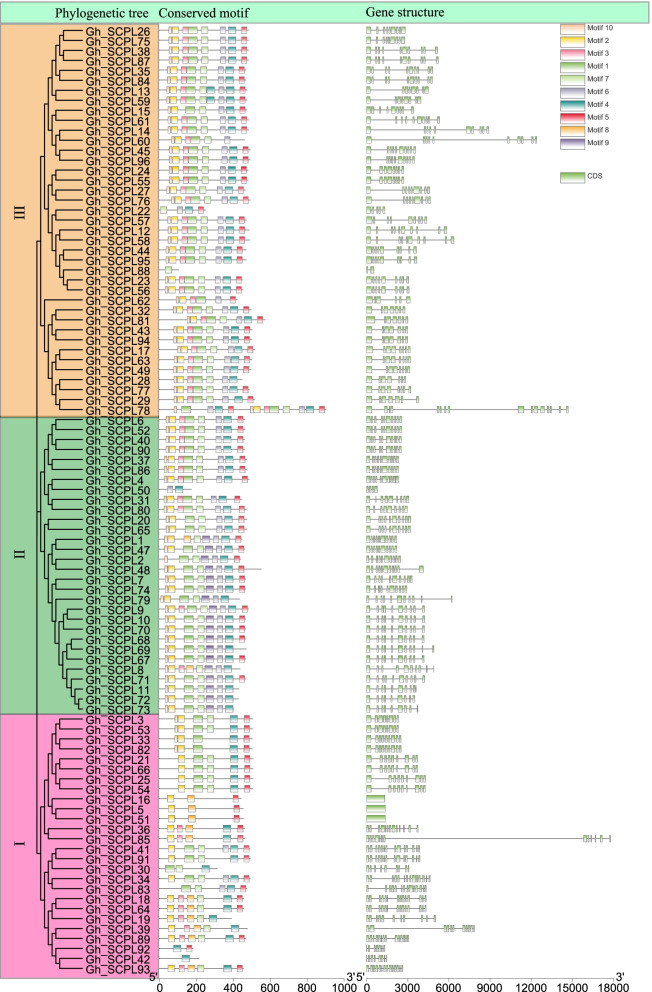
Evolutionary tree, gene structure and conservative motif analysis of SCPL gene family in *G. hirsutum*

The phylogenetic tree, gene structure and motif analysis were performed according to the full-length, CDS and protein sequences of the SCPL gene in *G. hirsutum* (Fig. 3). All members of SCPL gene family in *G. hirsutum* were divided into 3 subgroups according to the results of phylogenetic tree. Which were consistent with the findings in other crops. Among them, some genes in subgroup II contained motif9, while some genes in subgroup I contain motif8. Most of the members had the same motif (motif 2, 4 and 5) and contained 9/10 exons, indicating that the same family has similar functions. The gene motifs of subgroups II and III were very similar, indicating that these two subgroups might have originated from the tandem duplication of the same type of genes during evolution. *Gh_SCPL78* had the longest length and contained 5 repetitive motifs (motif1, 4, 5, 6 and 10) among SCPL genes in *G. hirsutum*. The difference between *Gh_SCPL78* and the gene structure of the same family might be the function of the gene had changed. It might be an error in genome annotation, which need further study.

### Analysis of cis-acting elements of the SCPL gene promoter in *G. hirsutum*

**Fig. 4:**
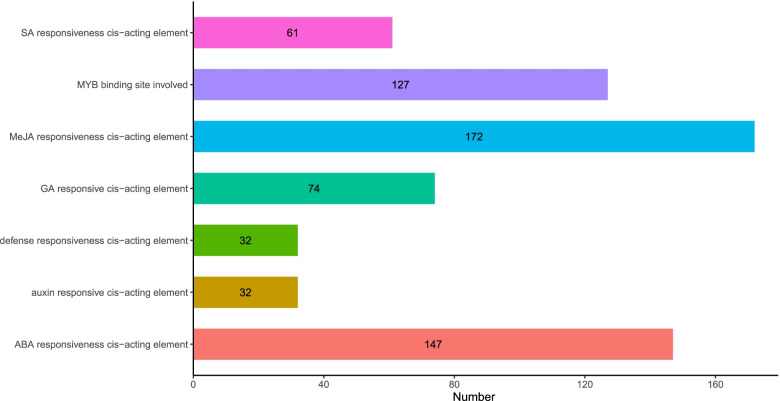
Quantitative analysis of cis-acting elements in the promoter region of SCPL family genes

Transcription factors (TFs) regulate plant functions, including responses to hormones and environmental factors, cell differentiation, and organ development by regulating gene expression [25]. Analysis of cis-elements in the promoter region of the SCPL gene in *G. hirsutum* revealed a variable number of cis-acting elements related to phytohormones and environmental stress (Fig. 4, Table S3). It mainly included cis-acting elements of defense, stress responses, salic acid, abscisic acid(ABA), gibberellin, auxin, jasmonic acid, and MYB binding sites. Each SCPL gene promoter contains different numbers and types of cis-acting elements in cotton, indicating that they might participate in different biotic and abiotic stress responses through different signaling pathways, MeJA and ABA ranked the most. It showed that the SCPL family might exert its biological functions mainly by cooperating with genes of MeJA and ABA biosynthesis or signal transduction in *G. hirsutum*.

### Expression analysis and genotyping of SCPL gene in *G. hirsutum*

**Fig. 5:**
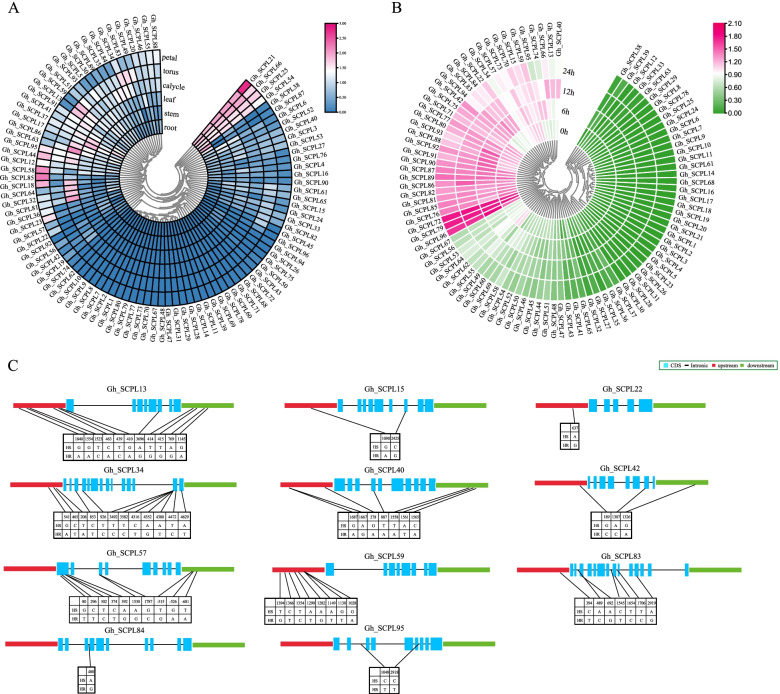
Expression analysis of SCPL genes in *G. hirsutum*. **A** the tissue-specific expression analysis, **B** the expression analysis at 0 h, 6 h, 12 h and 24 h under *Verticillium dahliae*, **C** SNP information between highly susceptible (HS) and highly resistant(HR)

The gene expression pattern is usually closely related to the function of the gene. In order to gain a deeper understanding of the expression pattern of the SCPL gene in *G. hirsutum*, Tissue-specific expression analysis of 96 SCPL genes and the expression pattern under the stress of Verticillium dahliae in *G. hirsutum* were conducted (Fig. 5). Tissue-specific analysis showed (Fig. 5A) that 64% of SCPL genes were expressed in different tissues, 4 genes (*Gh_SCPL21, 25, 54,* and *66*) were obviously expressed in roots, stems, leaves, receptacles, calyx, and petals.

In this study, the transcriptome data of roots infected with Verticillium dahliae in cotton were analyzed (Fig. 5B). The results showed that the expression of 16 genes (*Gh_SCPL13, 15, 22, 34, 40, 42, 57, 59, 66, 70, 73, 74, 83, 84, 94* and *95*) changed significantly within 0 ~ 24 h, 19 genes expressions(*Gh_SCPL-71, 72, 75, 76, 77, 79, 80, 81, 82, 85, 86, 87, 88, 89, 90, 91, 92, 93* and *96*) significantly higher than other genes, while the expression levels did not change significantly in each period. In order to further explore the candidate genes of resistance to Verticillium wilt in *G. hirsutum*, based on the resequencing results of 5-resistance and 5-susceptible extreme materials(Table S4). We typed 16 genes whose expression changed significantly before and after inoculation (Fig. 5C), and it was found that 11 of them (*Gh_SCPL-13, 15, 22, 34, 40, 42, 57, 59, 83, 84* and *95*) had differential SNPs in the upstream, downstream, exonic or intronic regions.

### qRT-PCR

**Fig. 6:**
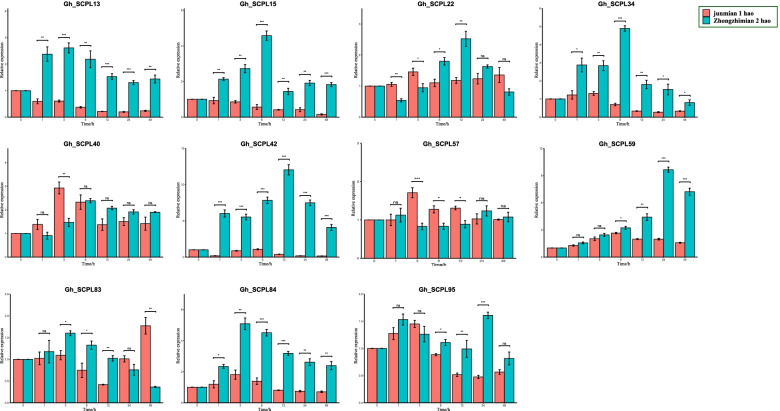
Expression analysis of SCPL gene in *G. hirsutum* under Verticillium dahliae. Error bars represent the average of three replicates ± SE. The difference with the control group is statistically significant, *P < 0.05, **P < 0.01, ***P < 0.001, respectively

Through the previous expression analysis combined with the gene typing results, we speculated that 11 genes (*GhSCPL-13, 15, 22, 34, 40, 42, 57, 59, 83, 84* and *95*) from SCPL gene might be involved in the defense of Verticillium wilt stress in *G. hirsutum*. qRT-PCR was used to detect the transcript levels of these 11 genes in resistant material (Zhongzhimian 2) and susceptible material (Junmian 1) (Fig. 6). Compared with 0 h, the RNA transcription levels of 6 genes (*GhSCPL-13, 15, 34, 42, 59* and *84*) in both materials were significantly induced at different periods under Verticillium wilt stress, and the expression levels were significantly increased, these genes might be involved in the process of response to Verticillium wilt stress in cotton. And in the same period, the expression of these genes in the resistant material was significantly higher than that in the susceptible material. In summary, these genes might play a role in the response of Verticillium wilt stress in *G. hirsutum*, rather than the natural high expression during the growth and development of cotton at this stage.

**Fig. 7:**
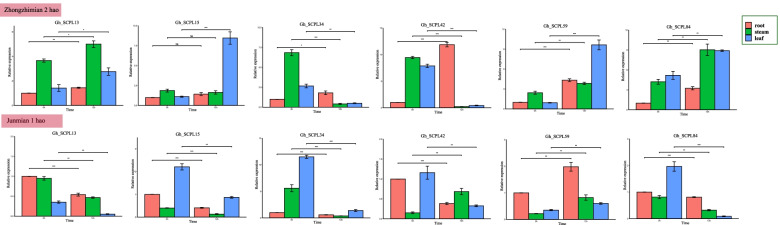
Tissue-specific expression analysis of SCPLgene in *G. hirsutum* under Verticillium dahliae. Error bars represent the average of three replicates ± SE. The difference with the control group was statistically significant, *P < 0.05, **P < 0.01, ***P < 0.001, respectively

Although previous study proved that the expression of these six genes change significantly in different periods after being infected with Verticillium wilt, and there were also significant differences in expression between materials. However, the molecular biological functions performed by gene expression are different depending on the tissue site of gene expression. To this end, the tissue-specific expression of these six candidate genes were further verified in two materials before and after 12 h inoculation. We found that there were significant changes in different tissues before and after inoculation (Fig. 7). Its initial sensing site was the root after infected with Verticillium dahliae in cotton, *Gh_SCPL42* had the most obvious change in the root. Results indicated that *Gh_SCPL42* plays an important role in the resistance of Verticillium wilt in *G. hirsutum*.

### VIGS validation of Gh_SCPL42 in cotton

**Fig. 8:**
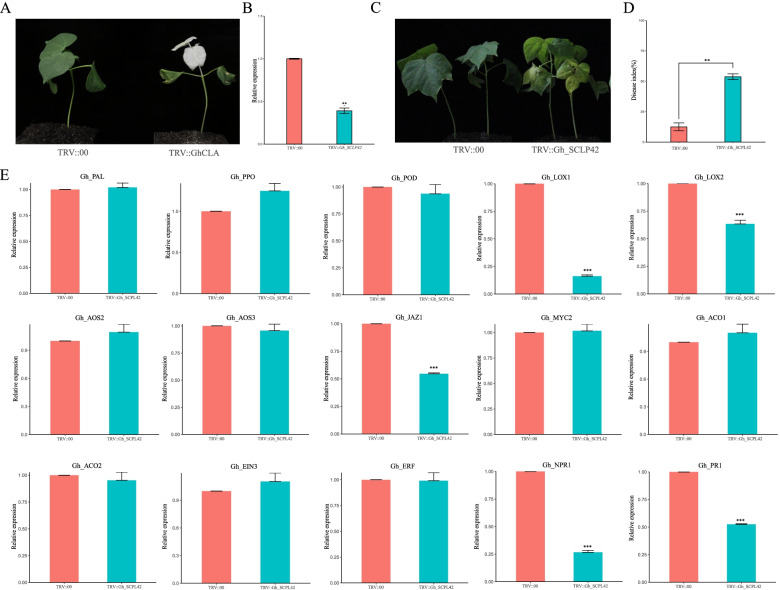
Effects of silencing of *Gh_SCPL42* on Zhongzhimian 2 susceptibility to V991. Two weeks after infiltration, seedlings were inoculated with V991. **A** Seven day-old cotton plants were infiltrated with Agrobacterium carrying TRV::*GhCLA*. The photographs were taken at 2 weeks after infiltration. **B** qRT-PCR for detection of silencing efficiency. **C** Representative seedlings of control (CK) and silenced plants after inoculation with V991 at 21 days post-inoculation (dpi). **D** Responses of control (CK) (*TRV::00*) and silenced (*TRV::Gh_SCPL42*) plants to the V991 at 21 dpi. Disease severity index (DSI) in CK and silenced plants each with 20 experimental replicates. The DSI were measured at 21 dpi. Error bars represent the standard deviation of 20 biological replicates; asterisks indicate statistically significant differences, as determined by t-test (*P < 0.05; **P < 0.01, ***P < 0.01). **E** qRT-PCR of genes related to SA, JA, ET and lignin pathway

To verify the function of *Gh_SCPL42* and analyze its molecular mechanism against Verticillium wilt. It was observed an albino phenotype on newly developed true leaves (Zhongzhimian 2) after injection of plants with Agrobacterium carrying *TRV1* + *GhCLA*, indicating that the virus induced gene silencing (VIGS) system was consistent with the expected results under experimental conditions (Fig. 8A). qRT-PCR analysis revealed that, the expression level of *Gh_SCPL42* in VIGS plants was significantly lower than that in the controlled plants (Fig. 8B). To further understand the relationship between *Gh_SCPL42* expression in VIGS plants and resistance of Verticillium wilt, the resistance to Verticillium wilt of VIGS plants were evaluated (Fig. 8C, D and Table S5). Compared with *TRV::00,* VIGS plants exhibited leaf necrosis and yellowing after inoculation (Fig. 8C) Significantly higher DSI and DSR was observed (Fig. 8D and Table S5). It was found that inhibition of the expression of *Gh_SCPL42* in Zhongzhimian 2 resulted in an increase in its susceptibility to Verticillium wilt.

Previous studies have found that salicylic acid (SA), jasmonic acid (JA), ethylene (ET), and lignin signaling pathways were the main signaling pathways of defense processes in cotton. The genes related to SA, JA, ET and lignin signaling pathways in *TRV2::Gh_SCPL42* and *TRV::00* plants was detected by performing qRT-PCR (Fig. 8E). The expression levels of genes related to lignin (*Gh_PAL, Gh_PPO* and *Gh_POD*) and ET (*Gh_ACO1, Gh_ACO2, Gh_EIN3* and *Gh_ERF*) did not change significantly before and after silencing, while the genes related to SA (*Gh_NPR1* and *Gh_PR*1) significantly down regulated. Among JA-related genes, *Gh_LOX1, Gh_LOX2* and *Gh_JAZ1* were also significantly down-regulated, while *Gh_AOS2, Gh_AOS3* and *Gh_MYC2* did not change significantly. It indicated that the regulatory mechanism of *Gh_SCPL42* on Verticillium wilt resistance was complex and needed further study. In conclusion, our findings suggested that *Gh_SCPL42* was a positive regulator of resistance to Verticillium wilt in *G. hirsutum*.

## Discussion

The SCPL gene family plays an important role in plant growth and development and resistance to stress [19–22]. Genome-wide analysis of SCPL genes in a variety of plants was performed by previous researchers. 71, 54, 57, 47, and 210 SCPL genes were identified in rice, *Arabidopsis thaliana*, poplar, tea, and wheat, respectively [8, 11, 23, 24]. These studies demonstrate that genome-wide identification and expression analysis can help researchers understand the origin, diversity, and biological function of these SCPL gene families. In recent years, with the improvement of sequencing technology and the continuous decrease of sequencing cost, the cotton genome sequencing work has been continuously improved and updated, which has laid the foundation for studying the function of genes from the whole genome level [5–8]. Further deepening the understanding of cotton genomics and genetics provides the possibility to explore the cotton SCPL gene family. At present, the research on cotton SCPL family genes has not been reported yet.

This study identified significantly more SCPL family genes in cotton than in rice, arabidopsis, poplar and tea for the first time, which may be the result of the doubling of the cotton genome, or it might be that more genes were annotated in cotton than in other species [5–8, 11, 23, 24]. Judging from the gene number, evolutionary tree and chromosomal location of the cotton SCPL gene family (Figs. 1 and 2B), in the long-term evolution of cotton SCPL, the SCPL gene family has always been a conserved family and is in line with the evolutionary relationship of cotton [5–8]. Protein structure and gene structure are closely related to gene function. Most genes in the same subgroup in the phylogenetic tree contained the same or similar gene structures and motif structures, which were consistent with those of model plants. In general, genes in the same subgroup exhibit similar exon/intron structure [25, 26], and changes in exon/intron structure or conserved domains may alter gene function [27]. Interestingly, the *Gh_SCPL78* gene contains more than 16 exons (Fig. 3), while none of the SCPL genes in rice, *Arabidopsis thaliana*, poplar, and tea contain as many exons [8, 11, 23, 24].

The genotyping method of resistant materials provides a strong basis for identifying candidate genes and lays a foundation for further molecular mechanism analysis. In this study, the differential GhSCPL genes were genotyped in combination with the transcriptome expression profile, and the differential SNP information was provided, laying a foundation for in-depth study of its molecular mechanism (Fig. 5C). We found SNPs upstream and downstream and in introns or exons of 11 SCPL genes, but most of the SNPs were located in the promoter regions or downstream regions of the genes, and there were relatively few SNPs in the CDS regions. This may indicate that the functional changes in SCPL tend to be regulated by other genes and that potential candidate genes can be identified at the expression level. Studies in maize, wheat and rice revealed that the expression levels of some functional genes did not change when plants were subjected to adversity stress, and there was no difference in extreme materials [28]. Although the possibility of the existence of the *G. hirsutum* SCPL gene is very small, it cannot be completely ruled out. Therefore, we screened the SCPL family genes of *G. hirsutum* by genotyping and qRT–PCR to identify potential candidate genes for Verticillium wilt resistance. We did not analyze the potential Indels; thus, we cannot rule out the possibility that other SCPLs in *G. hirsutum* may also have important functions in the process of resistance to Verticillium wilt. Gene expression patterns can provide important insights for characterization of gene functions, which are thought to be related to the differentiation of promoter regions, and transcription factors (TFs) play an important role in this process [29]. TFs regulate plant growth, development and stress resistance by regulating gene expression, mainly including responses to hormones and environmental factors, as well as cell differentiation and organ development [29]. Analysis of cis-elements in the promoter region of the SCPL gene showed that the most hormone-responsive elements in *G. hirsutum* were ABA and MEJA-responsive cis-elements, which was consistent with the findings in wheat. These results suggested that the SCPL gene family might be involved in plant responses to stress through ABA or MeJA pathways [8].

Wen et al. found that genes in the same family not only have redundant functions but also exhibit the same or different functions in the same biological process [30]. We found a similar situation via qRT–PCR regarding the expression after *G. hirsutum*. The RNA transcription levels of 6 genes (*GhSCPL13, 15, 34, 42, 59* and *84*) were significantly increased in both materials at different times, but only *Gh_SCPL42* changed most significantly in the roots. In contrast, the change in *Gh_SCPL13* was most obvious in the stem tissue. We speculate that the high expression of *Gh_SCPL13* in the vascular bundle may cause certain changes in the xylem or phloem during the invasion of cotton leaves by Verticillium wilt. The expression level of *Gh_SCPL34* in stem tissue was the highest before inoculation, but the expression level in stem tissue was the lowest at 12 h after inoculation, which indicated that *Gh_SCPL34* might be a negative regulator of upland cotton resistance to Verticillium wilt. Although the expression levels of these six genes in the roots, stems and leaves of *G. hirsutum* changed significantly before and after inoculation, much work is still needed in the future to produce transgenic cotton and to CRISPR/Cas9-mediated knockout in order to deepen understanding of the SCPL gene family. Validation of functions, discovery of important genes, and analytical verification of whether there is a functionally redundant relationship among SCPL genes in the resistance to Verticillium wilt are needed.

With the continuous deterioration of the global climate and the continuous planting of cotton, cotton production is subjecting to biotic and abiotic stress and constraints such as diseases, drought, salinity, etc. Continuous damages to cotton yield and fiber quality occurring [31–34]. The main goal of the current cotton breeding is to select new varieties with high yield and high quality that are resistant to Verticillium wilt. In this study, *Gh_SCPL42* was identified as a candidate gene for Verticillium wilt resistance by expression analysis combined with genotyping in cotton, and was silenced in cotton to study its role in cotton resistance to Verticillium wilt. We found that in the case of inoculated Verticillium wilt, silenced plants had severe disease and a significantly higher disease index than controls (Fig. 8B and C), By performing qRT-PCR, we found that *Gh_SCPL42* silenced plants in SA and JA-related pathway genes were significantly down-regulated (Fig. 8E). It was basically consistent with the function of *OsBISCPL1* [16]. Taken together, our findings suggested that *Gh_SCPL42*, as a positive regulator of Verticillium wilt resistance in *G. hirsutum*, the resistance to Verticillium wilt might be improved through the SA and JA pathways.

## Conclusion

In this study, the whole genome of cotton SCPL gene was identified. It was found that the SCPL gene family was conserved in the cotton evolution according to the number of genes, chromosomal location and evolution analysis. Expression analysis, genotyping and VIGS revealed that *Gh_SCPL42* was a positive regulator gene in response to Verticillium wilt stress in *G. hirsutum*. Our study was the first to systematically analyze the SCPL gene family, and provides a new understanding of the resistance to Verticillium wilt in cotton, which lays a foundation for the in-depth functional analysis and breeding application of *Gh_SCPL42*.

## Method

### Plant material

We selected Zhongzhimian 2 (disease resistance) and Junmian 1 (disease susceptibility) as the materials for expression analysis under Verticillium wilt stress. The inoculated Verticillium dahliae(V991) with highly virulent, deciduous. Zhongzhimian 2 and Junmian 1 were cultivated in soil culture. The middle and lower parts of the flowerpot were cut with a knife to ensure that the roots of the cotton are damaged at two true leaves and one heart stage, then the Verticillium wilt spore suspension (1 × 10^7^ spores mol/L) were applied near the roots of cotton seedlings, and each pot was inoculated with 50 mL of spore suspension. Three replicates of each material were planted, with 10 plants per replicate. At 0, 1, 3, 6, 12, 24 and 48 hpi (hours post infection), the roots, stems, leaves and other tissues of the two different materials treated with and untreated control were collected respectively.

### Identification and bioinformatics analysis of SCPL gene in cotton

The genomic and proteomic data of *G. arboreum*, *G. raimondii*, *G. hirsutum* and *G. barbadense* were obtained from COTTONGEN (http:/ /www.cottongen.org/) database. Protein sequences in cotton were identified by performing Hidden Markov Model of SCPL Gene Domain (PF00450) and Hidden Markov Model (HMM) in HMMER3.2.1 software (http://hmmer.org/) [35]. After removing redundancy, all candidate genes were verified in the NCBI-CDD (https://www.ncbi.nlm.nih.gov/cdd/) database, and the searched cotton SCPL gene family members were further confirmed [36]. ExPASy software (http://cn.expasy.org/tools) was used to calculate the number of amino acid residues, relative molecular mass, theoretical isoelectric point of SCPL protein in *G. hirsutum*. EuLoc online software (http://euloc.mbc.nctu .edu.tw/) was used for Subcellular localization prediction of SCPL protein in *G. hirsutum* [37].

### Phylogenetic and collinear analysis of cotton SCPL gene family

Using the default settings of Clustal W in MEGA X software, the SCPL protein sequences of *Arabidopsis thaliana*, *G. arboreum*, *G. raimondii*, *G. hirsutum* and *G. barbadense* were used for multiple sequence alignment (Gap opening penalty is set to 10, Gap Separation Distance is set to 4). Based on the results of the sequence alignment, the neighbor-joining method was used to build a phylogenetic tree with the Bootstrap value set to 1000 [38]. The resulting phylogenetic tree was beautified with the online tool Evolview (https://evolgenius.info/).

### Chromosomal location, gene structure and motif analysis of SCPL gene family in *G. hirsutum*

The chromosomal location information of SCPL gene family members was extracted from the *G. hirsutum* genome annotation file, and the chromosomal location map of SCPL gene was drawn by performing Mapchart software. The evolution tree was constructed for the SCPL in *G. hirsutum* by MEGAX software, and the nwk file was obtained. Motif analysis was conducted by MEME program (set the number of functional domains to 10) [39]. The xml file, the nwk file of the evolutionary tree and the gff file of the gene structure were processed and visualized by TBtools software [40].

### Analysis of upstream cis-acting elements of SCPL gene in *G. hirsutum*

The 2000 bp DNA sequence upstream of the SCPL gene in *G. hirsutum* was intercepted, and the possible cis-acting elements were predicted using the PlantCARE database (http://bioinformatics.psb.ugent.be/webtools/plantcare/html/), and visualized by using the R language ggplot package.

### RNA-seq analysis

Transcriptome data of organs (root, stem, leaf, pistil, stamen, calyx, petal and receptacle)under Verticillium wilt stress in *G. hirsutum* were downloaded from the NCBI SAR (Sequence Read Archive) database (Genome sequencing project accession: PRJNA248163 and PRJNA532694). Data filtering and quality control were performed with fastp software, and the resulting clean data were used for subsequent analysis [41]. The *G. hirsutum* TM-1 genome was used as a reference for read alignment (https://www.cottongen.org/species/Gossypium_hirsutum/ZJU-AD1_v2.1), and String Tie was applied to quantify the aligned reads, the FPKM method is used to assess gene expression [42–45]. The expression heat map was drawn with TBtools software [40].

### qRT-PCR analysis

According to the cDNA information in *G. hirsutum*, primers were designed at the specific region of gene sequence, 5′ or 3′ by using Primer 5.0 software (Table S6). The root tissue cDNA was used as the template, and the expression of candidate genes was detected by qRT-PCR. Each sample was repeated three times, and the internal reference gene was *GhUBQ7*.

qRT-PCR was performed as previously reported [46]. Total RNA extraction kit (Tiangen, China) was applied. Reverse transcription was performed using M-MLV RTase cDNA Synthesis Kit (TaKaRa, Japan). Real-time PCR amplification was performed with Applied Biosystems 7500 fast Real-time System. iTaq Universal SYBR Green Supermix (BioRad, USA) kit was used and according to the provided method, The amplification system was 20 μL the reaction program was pre-denaturation at 94 °C for 30 s, denaturation at 95 °C for 5 s, annealing at 60 °C for 5 s, and extension at 72 °C for 55 s, for 40 cycles. Relative quantification analysis was used by using the 2^–ΔΔCt^ method.

### VIGS

VIGS was performed in cotton based on tobacco rattle virus (TRV) [47]. A specific fragment of the *Gh_SCPL42* gene was inserted at the restriction site of the silencing vector pTRV2 to construct the silencing vector pTRV2-*Gh_SCPL42*, and pTRV2-*Gh_SCPL42* and pTRV2-*Gh_CLA* were transformed into Agrobacterium strain GV3101. Simultaneously, pTRV1 and pTRV2 empty vectors were also transformed into Agrobacterium strain GV3101. Primers used for amplification of the GhSCPL fragment are shown in Supplementary Table 6. Seven-day-old seedlings of Zhonghimian 2 were transformed with a mixture of Agrobacterium cultures containing pTRV1 and pTRV2 or plasmids. After the inoculation was completed, Zhongzhimian 2 seedlings were washed with deionized water to remove excess Agrobacterium inoculum and grown at 25 °C in an environmentally controlled growth chamber with a light/dark cycle of 16 h/8 h. After 2 weeks of culture, V991 was inoculated as previously described. Experiments were conducted with at least 20 seedlings per treatment and were repeated with 3 times. The morbidity index was assessed for each seedling.

## Supplementary Information

**Additional file 1.**

**Additional file 2.**

## Data Availability

The genome databases were downloaded from COTTONGEN (http://www.cottongen.org/) and RNA-seq (https://www.ncbi.nlm.nih.gov/sra/?term=). The genomic sequence datasets are downloaded from in the NCBI Sequence Read Archive under accession number PRJNA399050 (SRP115740). The datasets supporting the conclusions of this article are included in the article and its Additional files.

## Abbreviations

SCPL: Serine carboxypeptidase-like protein
MeJA: Methyl jasmonate
SA: Salicylic acid
TFs: Transcription factors
ABA: Abscisic acid
HS: Highly susceptible
HR: Highly resistant
VIGS: virus induced gene silencing
