# Supplementary material for: Genome-wide analysis of serine carboxypeptidase-like protein (SCPL) family and functional validation of Gh_SCPL42 unchromosome conferring cotton Verticillium der Verticillium wilt stress in Gossypium hirsutum
Source: BMC Plant Biol. 2022 Sep 1;22:421. doi: 10.1186/s12870-022-03804-5 (PMC9434971; doi:10.1186/s12870-022-03804-5)
Supplement: Supplementary file 1 — Additional file 1. [file 12870_2022_3804_MOESM1_ESM.pdf]

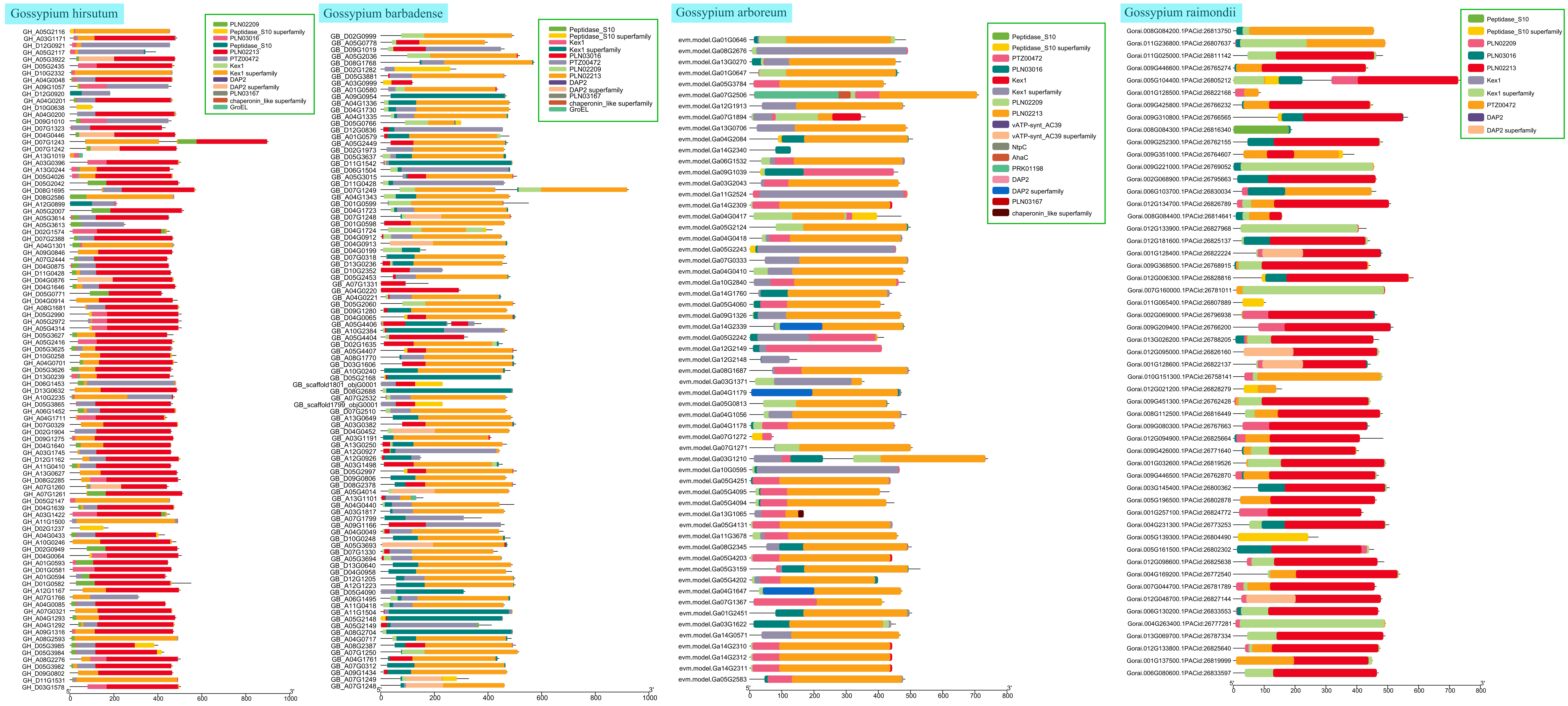

Figure S1.Distribution of the SCPL domain in the SCPL proteins of cotton.
